# Marker selection for whole-genome association studies with two-stage designs using dense single-nucleotide polymorphisms

**DOI:** 10.1186/1753-6561-1-s1-s136

**Published:** 2007-12-18

**Authors:** Jing Li

**Affiliations:** 1 Electrical Engineering and Computer Science Department, Case Western Reserve University, 10900 Euclid Avenue, Cleveland, Ohio 44106, USA

## Abstract

Large-scale genome-wide association studies are increasingly common, due in large part to recent advances in genotyping technology. Despite a dramatic drop in genotyping costs, it is still too expensive to genotype thousands of individuals for hundreds of thousands single-nucleotide polymorphisms (SNPs) for large-scale whole-genome association studies for many researchers. A two-stage design has been a promising alternative: in the first stage, only a small fraction of samples are genotyped and tested using a dense set of SNPs, and only a small subset of markers that show moderate associations with the disease will be genotyped in the second stage. In this report, I developed an approach to select and prioritize SNPs for association studies with a two-stage or multi-stage design. In the first stage, the method not only evaluates associations of SNPs with the disease of interest, it also explicitly explores correlations among SNPs. I applied the approach on the simulated Genetic Analysis Workshop 15 Problem 3 data sets, which have modeled the complex genetic architecture of rheumatoid arthritis. Results show that the method can greatly reduce the number of SNPs required in later stage(s) without sacrificing mapping precision.

## Background

A two-stage design has been a promising strategy for genome-wide association studies [1-5], primarily for the purpose of reducing genotyping costs. Studies have shown that two-stage designs can effectively reduce costs, even with a much higher per genotyping costs in stage two using specially designed arrays, compared to fixed arrays in stage one [3]. An optimal two-stage design to achieve a minimum cost with a similar overall significance level and statistical power depends on many factors such as disease allele frequencies, disease effects, fraction of samples genotyped in stage one, fraction of markers genotyped in stage two, as well as genotyping cost ratio in stage one and stage two. Several groups have investigated the issue using different statistical tests under different assumptions [1-4].

Generally speaking, there are three test strategies that can be adopted in stage two, namely, replication-based analysis, joint analysis assuming homogeneity between stages, and joint analysis that allows heterogeneity between stages [1-4]. In a replication-based study, data in stage two are considered alone and a positive association is reported if a statistical score reaches its significance level. In a joint analysis, subjects in stage one and in stage two will be considered together at the end, while raw data from two stages are combined first to obtain an overall statistic if assuming homogeneity, and statistics from two stages are combined if assuming heterogeneity [4]. A common practice to evaluate statistical significance for multiple tests by all three methods is to use Bonferroni adjusted *p*-values, which basically assumes all single-nucleotide polymorphisms (SNPs) are independent and in linkage equilibrium. Based on data from the HapMap project [6] and some other sources such as the Cancer Genetic Markers of Susceptibility (CGEMS) project , the assumption of linkage equilibrium is unlikely to hold when using SNP arrays with hundreds of thousands markers because many nearby SNPs are in high linkage disequilibrium. The Bonferroni correction is highly conservative and may partially explain the preliminary negative results from the CGEMS project: none of the 300 K SNPs are significantly associated with prostate cancer at a genome level of 0.05 after the Bonferroni correction. Permutations tests can be performed for the replication-based analysis, but it is not straightforward to extend permutation tests to joint analysis [7]. In addition, permutation tests are usually time-consuming and unlikely scale up to genome-wide studies. In this report, I explicitly explore the dependence between SNPs within a two-stage design using the simulated dense SNP data sets provided by Genetic Analysis Workshop 15 (GAW15) by applying a clustering algorithm and employing the joint analysis strategy for power studies.

## Methods

The algorithm was developed based on the following observations. For high-density SNP markers (e.g., 300 K or 500 K SNP arrays), it is likely that nearby SNPs are in linkage disequilibrium (LD). In a two-stage design, usually a liberal significance level *α *(such as 0.05 without the Bonferroni correction) in stage one is used to ensure that no true signals will be filtered out. On average, *Mα *SNPs will be selected to stage two, where *M *is the total number of markers in stage one which is 300 K or 500 K. However, most of the *Mα *SNPs are false positives with respect to the disease in study. Furthermore, if a SNP shows a moderate association with the disease and has been selected in stage one, it is highly likely that its nearby SNPs that are in high LD with it will also be selected to stage two. In other words, many of the *Mα *SNPs may also have high LD. Therefore, I propose to apply a clustering algorithm to all SNPs that have been selected from stage one to explore the dependence relationship among the *Mα *SNPs. More specifically, all the *Mα *SNPs are first ranked according to their significance levels. Starting from the SNP with the highest rank (smallest *p*-value), all of the SNPs that are highly correlated with it (with the pairwise LD D' larger than a predefined threshold) will be grouped as a cluster conditional on the requirement that they are within a certain physical distance (which is a parameter). The cluster will be represented by the SNP with the highest rank. The process will continue in the decreasing order of SNP ranking for all SNPs that have not yet been clustered, until all the SNPs have been processed. At the end, the algorithm returns a set of clusters, each represented by a SNP with the highest rank within its cluster. A SNP can only be grouped to a nearby representer (defined by the distance threshold) to eliminate false signals of LD that can occur between two SNPs by chance. SNPs in a cluster are not necessarily consecutive. Clearly, the above clustering algorithm can reduce the number of SNPs to be considered in stage two and its effectiveness depends upon correlations among SNPs, as well as the two parameters. Joint analysis assuming heterogeneity is adopted in this study because it has higher power than replication-based analysis and it requires fewer assumptions. A proper significance level has to be derived for such an analysis. In general, suppose a liberal significance level *α *with the critical value *c*_1 _is used in stage one. Let *X*_1 _denote the *χ*^2 ^test statistic based on samples in stage one. Only markers with *X*_1 _> *c*_1 _will be further considered in stage two. For a marker to be genotyped in stage two, let *X*_2 _denote the test statistic using samples from stage two. Under the null hypothesis of no association, *X*_1 _and *X*_2 _are independent and follow *χ*^2 ^distributions with 1 degree of freedom. For the joint analysis, the statistic *X *is equal to the summation of *X*_1 _and *X*_2_. Notice that *X *and *X*_1 _are not independent even under the null distribution. Let *f*(*x*) and *F*(*x*) denote the probability density function and the cumulative distribution function of *χ*^2 ^distribution with 1 degree of freedom, the significance level of *X *with a value *c *can be calculated based on the following formula through numerical methods:

$$P(X>c|X_{1}>c_{1})=\left\{\int_{c_{1}}^{c}(1-F(c-x_{1}))f(x_{1})dx_{1}+(1-F(c))\right\}/\alpha .$$

I applied the above clustering algorithm within a two-stage design using the joint analysis on the simulated data sets of Problem 3. All analyses were carried out with knowledge of true disease gene locations. I first tested the above algorithm on the dense SNP set on chromosome 6, which contains the *HLA-DRB1 *locus and Locus D. The total number of SNPs is 17,820, with an average inter-marker interval of 10 kbp, which corresponds to a 300 K array. As a comparison, I also applied the algorithm on the SNP data of chromosome 18 that mimic a 10 K SNP chip set. SNP data on chromosome 1 were used to evaluate the type I errors. I first constructed data sets for a case-control study with a two-stage design. For each data set, only one affected child was randomly chosen as a case subject from each nuclear family with an affected sib pair. One child is selected as a control subject from each normal family. Therefore, all cases and controls are independent. Because some alleles around the *HLA-DRB1 *locus have very strong effects on the disease status, only a very small fraction of cases and controls were randomly selected for testing from all subjects (1500 cases and 2000 controls). Let *n *denote the total number of subjects tested in stage one and stage two together, where an equal number of cases and controls were tested. For chromosome 6, *n *took the values of 100, 200, and 300. Let *f *denote the fraction of the number of subjects in stage one, and *f *took the values of 0.3, 0.4, and 0.5 in this experiment. I assumed only *nf *subjects were genotyped for all *m *SNPs in stage one. The Pearson *χ*^2 ^statistic was used to select a subset of *k *SNPs for stage two based on a significance level of 0.05 without adjustments. The clustering algorithm was then applied to the *k *SNPs with a LD threshold D' = 0.8 and a distance threshold of 100 kbp for chromosome 6. For each parameter combination, 100 independent replicates were randomly sampled from the original data sets. I have investigated and compared the power, costs, significance levels, and prediction errors (the distances from the predicted locations to the true gene location) of three methods, namely, the one-stage design using all data, the two-stage design without clustering, and the two-stage design with clustering. For chromosome 18 and chromosome 1, because the total number of markers on each chromosome is much smaller than the number of SNPs on chromosome 6, and the effect of Locus E on chromosome 18 is much smaller than the *HLA-DRB1 *locus, a different set of parameters has been used (e.g., *n *= 750, 1000, 1250; and the distance threshold for clustering is 5 Mbp).

## Results

### Power, number of positive SNPs, and significance levels

Because of the unusually strong effect of the *HLA-DRB1 *locus, all three methods have returned more than one significant SNPs that are close to the locus, even with as few as 100 individuals (Table 1). The numbers of positive SNPs increase dramatically with the increase in sample sizes, while show little decrease when using a more stringent overall significance level. Most of the SNPs are not causal SNPs but are in close linkage and high association with causal SNPs. A few that are far from the causal SNP can be regarded as false positives. With clustering, the number of positive SNPs drops to half to one-third of the number without clustering, which indicates that the clustering algorithm has grouped many SNPs selected from stage one together because they are close to each other (distance < 100 kbp) and have high correlations (D' ≥ 0.8). Because it is impossible to directly assess power of the three methods using current data sets, the significance levels of the most significant SNPs by three methods are presented in Table 2. The one-stage design achieves the most significant results (smallest *p*-values), even after being adjusted by the number of total tests. The power of joint analysis with two-stage designs is close to that of the single stage design. The two-stage design with clustering achieves slightly better results than the original two-stage design.

**Table 1:** Mean (SD) number of positive SNPs at significance level *α *and fraction of samples *f *in stage one and for sample sizes 100, 200, and 300 for each method (one-stage design, two-stage design, and two-stage design with clustering)

		100			200			300		
				
*α*	*f*	1 stage	2 stage	2 stage-c	1 stage	2 stage	2 stage-c	1 stage	2 stage	2 stage-c
0.05	0.3		15.8(± 0.42)	6.8(± 0.20)		27.6(± 0.54)	10.7(± 0.25)		39.7(± 0.62)	15.0(± 0.29)
	0.4	17.9(± 0.44)	16.1(± 0.43)	6.5(± 0.19)	31.1(± 0.62)	28.3(± 0.58)	11.3(± 0.25)	44.9(± 0.71)	40.1(± 0.63)	15.3(± 0.27)
	0.5		16.1(± 0.42)	6.9(± 0.18)		28.2(± 0.56)	11.4(± 0.26)		40.5(± 0.64)	15.7(± 0.27)
										
0.01	0.3		14.0(± 0.41)	6.0(± 0.20)		24.6(± 0.48)	9.8(± 0.23)		35.6(± 0.57)	13.5(± 0.27)
	0.4	15.7(± 0.41)	14.2(± 0.38)	6.0(± 0.18)	27.6(± 0.54)	25.0(± 0.47)	10.4(± 0.22)	39.4(± 0.62)	36.0(± 0.54)	14.0(± 0.26)
	0.5		14.2(± 0.39)	6.2(± 0.16)		25.1(± 0.48)	10.3(± 0.23)		36.1(± 0.56)	14.3(± 0.25)

**Table 2:** Mean (SD) significance levels (-log_10_(*p*)) for each design for fraction of samples *f *in stage one and for sample sizes 100, 200 and 300 for each method

	100			200			300		
			
*f*	1 stage	2 stage	2 stage-c	1 stage	2 stage	2 stage-c	1 stage	2 stage	2 stage-c
0.3		10.8(± 0.29)	11.0(± 0.30)		25.1(± 0.40)	25.3(± 0.41)		39.7(± 0.47)	39.9(± 0.49)
0.4	11.6(± 0.30)	10.7(± 0.29)	11.0(± 0.30)	26.1(± 0.40)	25.1(± 0.40)	25.4(± 0.40)	40.8(± 0.47)	39.7(± 0.47)	40.1(± 0.47)
0.5		10.8(± 0.30)	11.1(± 0.30)		25.2(± 0.40)	25.5(± 0.40)		39.7(± 0.47)	40.1(± 0.47)

### Distances

Another measure to compare the three methods is to look at the distances of the predicted locations (the most significant SNPs) from the location of the *HLA-DRB1 *locus. Interesting, there are no significant differences between the three methods (Table 3). Although the effect of *HLA-DRB1 *locus is so strong, the most significant SNPs can be located 70 kbp away. The two-stage method with clustering can significantly reduce the number of typed SNPs in stage two without losing any precision in terms of mapping utility.

**Table 3:** Mean (SD) distances of the predicted locus from the disease locus (kbp) for fraction of samples *f *in stage one and for sample sizes 100, 200 and 300 for each method

	100			200			300		
			
*f*	1 stage	2 stage	2 stage-c	1 stage	2 stage	2 stage-c	1 stage	2 stage	2 stage-c
0.3		68(± 5.8)	85(± 5.8)		85(± 6.0)	90(± 5.9)		82(± 5.7)	83(± 5.7)
0.4	68(± 5.7)	67(± 5.7)	75(± 5.8)	86(± 6.0)	87(± 6.0)	91(± 6.0)	81(± 5.6)	81(± 5.6)	82(± 5.7)
0.5		69(± 5.8)	73(± 5.9)		86(± 6.0)	90(± 6.0)		80(± 5.7)	81(± 5.7)

### Number of genotyped SNPs and costs

By clustering nearby SNPs that are in high LD, one can significantly reduce genotyping costs in stage two. On average, the number of SNPs for the second stage with clustering (781 ± 12) is only about one half of the number of SNPs without clustering (1846 ± 72). Those numbers are very robust with regard to sample sizes and the fractions of samples being genotyped in stage one. The costs for the two methods with a two-stage design are the same for stage one (which is about half to 30% of the cost of one-stage design). And the cost of the two-stage design with clustering is about half of it without clustering. The overall saving depends on the cost ratio of genotyping a single SNP in stage one and in stage two.

### Rare alleles

There is another locus on chromosome 6 about 5 cM away from the *HLA-DRB1 *locus that contributes to the development of rheumatoid arthritis (RA). But the disease allele has a very low frequency (0.0083) the above procedure cannot detect the signal with small sample sizes (smaller than 300).

### Results on chromosome 18

The same procedure has been applied on chromosome 18 (with 303 markers) using a different set of parameters. Results show that almost no SNPs that are significant in stage one can be grouped together when the LD threshold D' = 0.8, even when the distance threshold as large as 5 Mbp. Therefore, the above approach is effective when using very dense SNP sets such as 300 K or 500 K arrays.

### Type I errors

No genes on chromosome 1 have effects on RA in the simulated data, so it was taken as a data set in evaluating type I errors for the three methods. Because this is another data set mimicking a 10 K SNP chip, the results from the two-stage designs with and without clustering are quite similar, and both methods have correct type I errors at both 0.05 and 0.01 level (sample sizes 750, 1000 and 1250). The one-stage design using Bonferroni correction has correct but much lower error rates, which means a Bonferroni correction is conservative even for SNPs with low correlations.

## Discussion and conclusion

For very dense SNP arrays, it is highly likely that SNPs within a short distance are not independent from each other. In this report, I have investigated a strategy of evaluating SNP correlations within a two-stage design using case-control samples, and have applied the algorithm on the Problem 3 simulated data sets of GAW15. The strategy can reduce the genotyping costs in stage two by half with similar or better performance (power/significance level, number of false positives, mapping precision) on data sets based on 300 K SNP arrays. Two-stage designs are promising for genome-wide association studies. As illustrated in this paper, advanced processing in stage one can further reduce genotyping costs in later stages without sacrificing mapping precision. A potential drawback using SNPs with little redundancy is that a failed assay in stage two for a marker SNP will lose information on a whole region of the genome.

## Competing interests

The author(s) declare that they have no competing interests.
